# A Study of the Localized Ceria Coating Deposition on Fe-Rich Intermetallics in an AlSiFe Cast Alloy

**DOI:** 10.3390/ma14113058

**Published:** 2021-06-03

**Authors:** Salil Sainis, Caterina Zanella

**Affiliations:** 1 Department of Materials and Manufacturing, School of Engineering, Jönköping University, Gjuterigatan 5, 55111 Jönköping, Sweden; caterina.zanella@ju.se; 2 Department of Industrial Engineering, University of Trento, via Sommarive 9, 38123 Trento, Italy

**Keywords:** conversion coating, cerium, localized deposition, Fe, intermetallic, SEM, EDS, AFM, SKPFM, microstructure-induced localized deposition

## Abstract

Corrosion inhibiting conversion coating formation is triggered by the activity of micro-galvanic couples in the microstructure and subsequent local increase in pH at cathodic sites, which in the case of aluminium alloys are usually intermetallics. Ceria coatings are formed spontaneously upon immersion of aluminium alloys in a cerium conversion coating solution, the high pH gradient in the vicinity of intermetallics drives the local precipitation of ceria conversion compounds. Cu-rich intermetallics demonstrate a highly cathodic nature and have shown the local precipitation reaction to occur readily. Fe-rich intermetallics are, however, weaker cathodes and have shown varied extents of localized deposits and are in focus in the current work. Model cast Al-7wt.%Si alloys have been designed with 1 wt.% Fe, solidified at different cooling rates to achieve two different microstructures, with big and small intermetallics, respectively. Upon subjecting the two microstructures to the same conversion coating treatment (immersion in a 0.1 M CeCl_3_ solution) for a short period of 2 h, preferential heavy deposition on the boundaries of the big intermetallics and light deposition on the small intermetallics was observed. Based on these observations, a mechanism of localized coating initiation at these Fe-rich intermetallic particles (IM) is proposed.

## 1. Introduction

Cerium-based conversion coatings (CeCC) have shown promise due to their effective corrosion protection [1,2], environmental compliance, and relative abundance of the raw material [3]. The corrosion inhibition imparted to aluminium alloys upon their immersion in cerium chloride salts was discovered in 1984 at the Aeronautical Research Laboratory in Australia by Hinton et al. [4,5,6,7]. Since then, several works have focused on understanding the various parameters affecting the deposition, reviewed extensively by Harvey [8]. The inhibition mechanism involves spontaneous deposition, upon immersion in conversion-coating solutions, of conversion compounds like cerium oxide and hydroxide [7] that form a barrier layer over the substrate and subsequently prevent contact with the electrolyte. The spontaneous deposition is driven by micro-galvanic coupling-induced local rise in pH, which is a consequence of oxygen reduction reaction at relatively cathodic sites of the microstructure. While the driving force for their deposition is a consequence of an electrochemical oxygen reduction reaction, the formation of these coatings is a precipitation reaction resulting from the reaction of cerium and hydroxide ions in solution. The formation of CeCC are explained based on the island growth hypothesis [4,9], where precipitates initiate at cathodic intermetallics showing an island-like coating structure that subsequently grows to cover the entire alloy surface.

The most investigated substrates for spontaneous coating deposition are Cu-containing AA2024 and AA7075 alloys. The presence of Cu-rich intermetallic particles (IM), which have high cathodic activity, enhances the precipitate deposition reaction and several works have investigated the deposition mechanism on them [3,4,10,11,12,13,14,15]. The consensus is that conversion compounds first precipitate preferentially on Cu-rich IM due to high pH gradient just over it and these localized deposits grow to cover the entire surface. Other non-Cu containing alloys, also with cathodic intermetallics albeit of lower cathodic potential, have proved challenging to coat. While researchers have pointed out that heavy precipitate products are observed on Cu-rich IM due to their highly cathodic nature, their presence in the microstructure is not essential [16,17]. The success in coating non-Cu containing IM, however, is varied.

Fe is an inevitable impurity found in the composition of most aluminium alloys, arising from either the ore or from primary aluminium refining and can be introduced during the recycling of aluminium or during the casting process [18]. Fe thus manifests into different IM that are found in the various aluminium alloys. While they are cathodic relative to the aluminium matrix, localized deposition of CeCC is not always guaranteed upon immersion of aluminium alloy in a cerium conversion coating solution. In work by Isaacs et al. [19], Cu-rich IM were preferentially covered with a deposit but IM of Fe/Mn were not. Hughes further explained that inhomogeneous film with varying thickness over different intermetallics, especially thick over Cu-rich and very thin over Fe-Mn intermetallics, was due to their varying cathodic reduction efficiencies. Pardo et al. [20] has observed localized CeCC deposition occurring on β-(Al,Fe,Si) IM and Al–Fe–Si–Mn IM in the cast alloy A361/SiCp composite. Eslami et al. [21] have also observed coating to initiate at the Fe-rich IM in a Al–Si–Fe alloy, but when cast Al–Si alloys contained both Cu and Fe, preferential heavy deposition was seen only on Cu-rich IM as shown in a study by Sainis et al. [22]. Other works [23,24,25] have shown varying degrees of surface coverage and the imparted corrosion resistance.

In this study, an effort has been made to investigate the mechanism of deposition of these difficult-to-coat Fe-rich IM. Model cast Al–Si alloys have been designed that have only additional Fe as the alloying element, to allow the formation of only Fe-rich IM in the microstructure. To additionally investigate the influence of size on the localized deposition mechanism, “coarse” and “fine” microstructures have been synthesized by subjecting the alloy to different solidification rates. The coarse and fine alloys contain big and small Fe-rich IM respectively. The microstructure, morphology and local electrochemical properties of the bare IM and localized CeCC deposits on them obtained from the conversion coating treatment have been characterized and analyzed in this study.

## 2. Materials and Methods

Sample preparation—A model alloy with composition shown in Table 1 was selected and subjected to directional solidification. Cross-sectional discs (Ø10 mm × 10 mm) were cut from two different directionally solidified cylindrical specimens and mounted in a conductive Struers PolyFast resin of size Ø30 mm × 20 mm. They were then ground with SiC papers up to P4000, followed by polishing with diamond suspension solution of colloidal particle sizes down to 0.25 µm. They were then rinsed in an acetone bath for 10 min to remove fine residues from polishing and to degrease the surface.

Surface preparation before coating treatment—the surfaces in this study were activated for deposition by etching away the native aluminium oxide in a 2 wt.% or 0.5 M NaOH solution for 2 min and then rinsed with distilled water.

Coating deposition—the samples were mounted in a laboratory manufactured deposition cell where only the metallic surface was exposed to the conversion coating solution. The deposition cell had a 50 mL reservoir with 8 mm diameter surface exposure. Such a set up was utilized to expose only the alloy surface and to avoid any unwanted electrochemical effects of the conductive resin. The conversion coating solution, 0.1 M CeCl_3_, was prepared fresh and filled into the deposition cell reservoir after mounting the sample. The alloy surfaces were exposed to the conversion coating solution for three different periods of 1 h, 2 h and 4 h. After these exposure times, the samples were rinsed with distilled water and placed in a desiccator to dry.

Surface Characterization—A JOEL JSM-7001F (Cedarburg, WI, USA) scanning electron microscope (SEM) in secondary electron (SE) imaging mode at 15 kV accelerating voltage was used for characterizing the morphology of the etched and deposited surfaces. Furthermore, an in-built energy-dispersive X-ray spectroscopy (EDS) (EDAX Apollo X SDD) detector was utilized for elemental distribution.

Surface topography of the etched surface and localized deposits was performed using a Park Systems NX10 atomic force microscope (AFM) in non-contact mode. Scanning Kelvin probe force microscopy (SKPFM) using the same system was utilized to characterize the local electrochemical properties of the intermetallics as well as deposits.

## 3. Results and Discussion

### 3.1. Microstructure of Directionally Solidified Cast Al–Si–Fe Alloys

**Figure 1:**
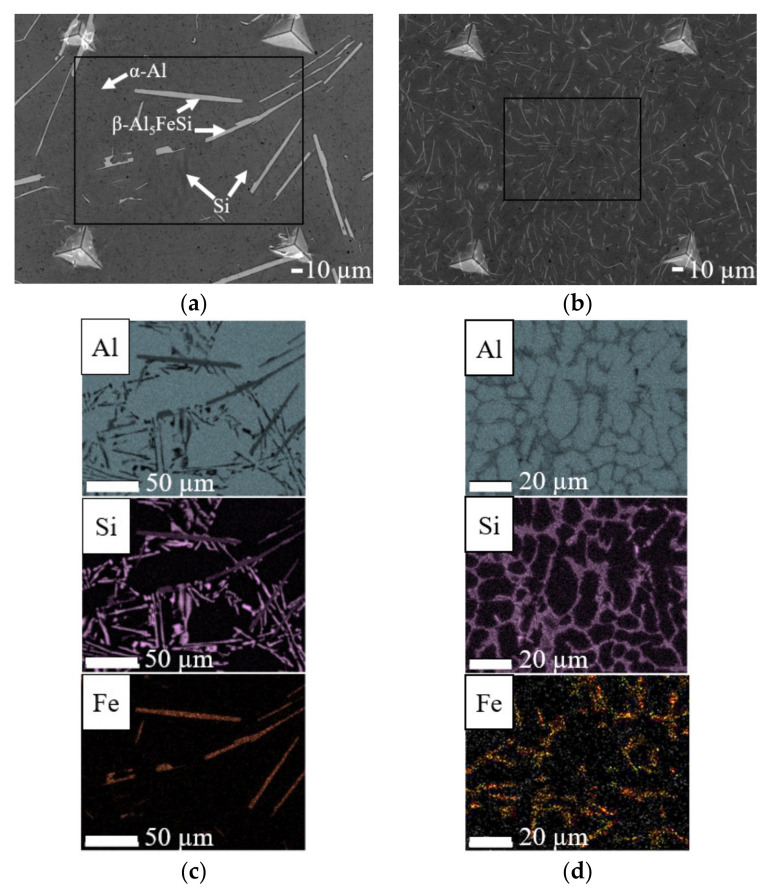
Characterization of microstructure morphology with scanning electron microscopy in secondary electron imaging mode (SEM-SE) and elemental distribution with energy-dispersive X-ray spectroscopy (EDS) of (**a**,**c**) coarse and (**b**,**d**) fine Al–Si–Fe alloys.

Kinetics of solidification determine whether a “coarse” or “fine” microstructure (Figure 1a,b) is achieved and to obtain them, slow (0.03 mm/s) and fast (6 mm/s) directional solidification rates have been applied respectively. Both microstructures consist of primary aluminium (α-Al), Al–Si eutectic and plate-type IM β-Al_5_FeSi within the eutectic region. The IM has been identified through comparison of the morphology of IM in the microstructure with those in literature [26,27], XRD characterization [28] and EDS elemental area mapping (Figure 1c,d). The IM of interest in the coarse microstructure are 20–100 µm long and 2–10 µm wide whereas the fine IM are 1–20 µm long and 0.1–1 µm wide.

**Figure 2:**
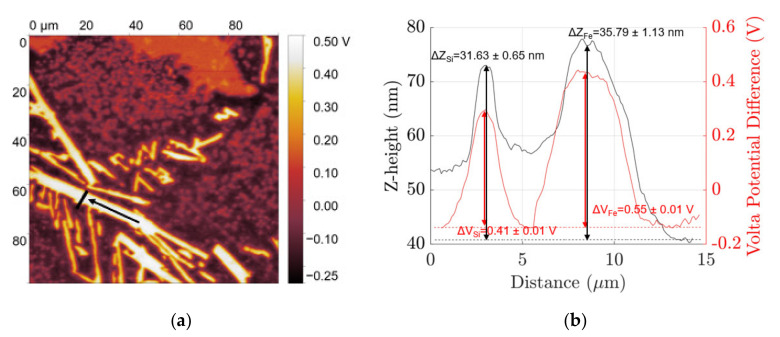
(**a**) Scanning Kelvin probe force microscopy (SKPFM) map of freshly polished coarse microstructure and (**b**) volta potential (red) and z-height (black) distribution profile along the line indicated in the SKPFM map.

**Figure 3:**
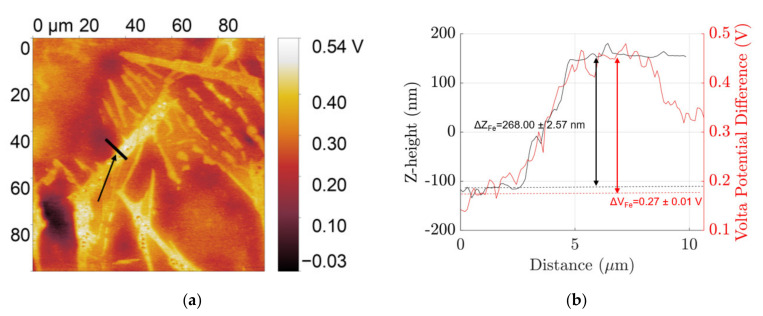
(**a**) SKPFM map of NaOH etched coarse microstructure and (**b**) volta potential (red) and z-height (black) distribution profile along the line indicated in the SKPFM map.

**Figure 4:**
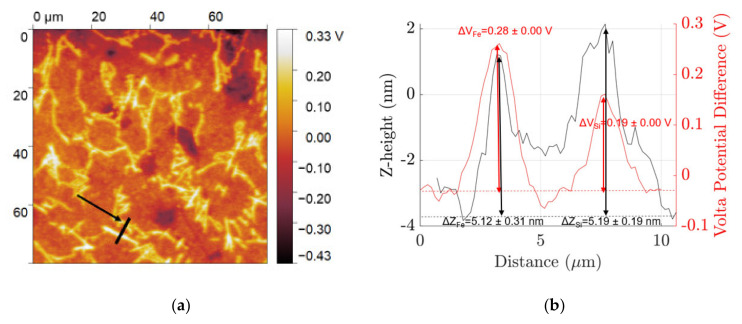
(**a**) SKPFM map of freshly polished fine microstructure and (**b**) volta potential (red) and z-height (black) distribution profile along the line indicated in the SKPFM map.

**Figure 5:**
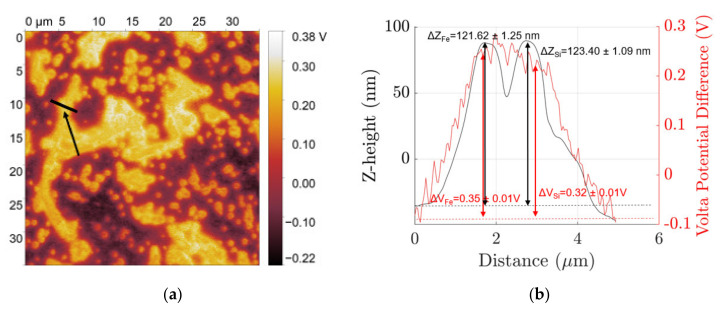
(**a**) SKPFM map of NaOH etched fine microstructure and (**b**) volta potential (red) and z-height (black) distribution profile along the line indicated in the SKPFM map.

AFM-SKPFM have been used to characterize the topography and volta potential of the different phases in the microstructure of polished and etched surfaces of coarse Al–Si–Fe alloy in Figure 2 and Figure 3 and fine Al–Si–Fe alloy in Figure 4 and Figure 5, respectively. The coarse β IM and Si particles from the Al–Si eutectic protrude out from the surface of at z-heights +35.79 ± 1.13 nm and +31.63 ± 0.65 nm respectively relative to the Al matrix, mainly due to their varying hardnesses. These particles in the polished condition show a volta potential difference relative to the aluminium matrix, ΔV_Fe_ and ΔV_Si_, of +0.55 ± 0.01 V and +0.41 ± 0.01 V, respectively, showing their cathodic nature. When etched in 0.5 M NaOH solution for 2 min, which dissolves the native aluminium oxide layer as well as some of the Al matrix, the β IM protrude out +256.00 ± 2.57 nm. Furthermore, SKPFM characterizations show a cathodic nature with +0.4 V volta potential difference relative to the aluminium matrix.

In the mechanically polished surface, fine β IM and Si particles protrude out +5.12 ± 0.31 nm and +5.19 ± 0.19 nm, respectively, from the aluminium matrix due to differences in hardnesses and show a cathodic nature with ΔV_Fe_ = +0.28 ± 0.01 V and ΔV_Si_ = +0.19 ± 0.01 V. After etching, each of their z-height relative to the matrix is +121.62 ± 1.25 nm and 123.40 ± 1.09 nm, respectively, for β IM and Si with ΔV_Fe_ = +0.35 ± 0.01 V and ΔV_Si_ = +0.32 ± 0.01 V.

### 3.2. Localized Deposition of Cerium Compounds upon Conversion Coating Treatment

**Figure 6:**
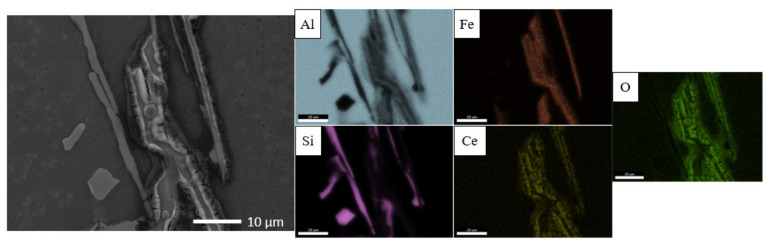
Morphology and elemental distribution of conversion coated coarse microstructure after immersion in 0.1 M CeCl_3_ for two hours.

Localized deposition of cerium conversion compounds is driven by micro-galvanic couple-induced local-pH-rise [4,7,8] at cathodic sites and thus deposition is expected to occur at both β IM and Si particles due to their relative positive ΔV relative to the aluminium matrix. However, it must be noted that CeCC precipitates as cerium oxides/hydroxides only at a high enough pH [8] measured with tungsten microelectrode to be around 8.5 in a study by Li et al. [29]. Figure 6 shows the morphology and elemental distribution maps of deposition on coarse microstructure after conversion coating treatment in 0.1 M CeCl_3_ solution for 2 h. Preferential heavy cerium compound deposition, possibly cerium oxide and hydroxide [7], was observed on the Fe-rich β IM but not on Si particles despite their similar volta potential difference relative to the Al matrix. The localized coating deposit has a “cracked-mud” morphology, typical characteristic of cerium conversion coatings reported in several other articles [3,14,19,30,31]. From looking at the EDS elemental distribution maps in Figure 6, it is evident that the cracked-mud morphology deposits on Fe-rich IM is a compound of Ce-O. Neither Ce nor O signal was obtained from the Si particles.

Si particles have been shown to not support a spontaneous deposition reaction [20,22] upon immersion in conversion coating solution due to their low conductivity [32]. Furthermore, Pardo et al. [20] have shown that even upon cathodic polarisation of cast A3xx.x/SiCp composite microstructure in a conversion coating solution, lesser deposition was observed on SiCp due to the low efficiency of electron transfer from Si. The hindrance to the transfer of electrons from the Si phase to local O_2_ in solution hinders the spontaneous local reduction of oxygen and, therefore, no cerium conversion coating precipitation reaction to occur.

Studies that have investigated conversion coating deposition mechanisms on Fe-rich IM, have implemented long conversion coating times upwards of 18 h [21,30]. At prolonged conversion coating times, the kinetically slow precipitation process has sufficient time to form an island-type deposit covering the whole IM. In the current study, however, we have implemented a shorter conversion coating time of 2 h to capture the initial stages of precipitation. It appears that the initiation of conversion compound precipitates occurs at the interface of the Fe-rich IM β and Al matrix, as can be seen from Figure 6, where localized deposits were observed to form only at the boundary of the Fe-rich IM β. In a similar study, ref. [22] investigating localized deposit initiation on a mixed microstructure containing Fe-rich IM β as well as Cu-rich IM θ-Al_2_Cu and ω-Al_7_Cu_2_Fe, preferential heavy deposition was seen only on Cu-rich IM upon immersion of the alloy in a solution containing Ce^3+^ ions. Even at shorter immersion times of 0.5 h, the whole Cu-rich IM showed a heavy deposit, not just at the boundaries. Cu-rich IM show a more cathodic nature and are able to support rapid oxygen reduction reactions and consequently localized deposition.

However, it must be noted that the type of deposition shown on Fe-rich IM in Figure 6 was not observed on all the coarse Fe-rich IM of the microstructure. possibly indicating that deposition either did not occur, or more likely, a very thin layer of conversion coating not visible by SEM was formed due to the kinetics of precipitation reaction being slow. It was shown in a Cu-rich IM containing microstructure that the percentage number of Cu-rich IM covered with a localized deposit increased with increasing conversion coating time. Due to short immersion time implemented in the current study, it is likely that not all Fe-rich IM were activated for preferential heavy deposition on them.

**Figure 7:**
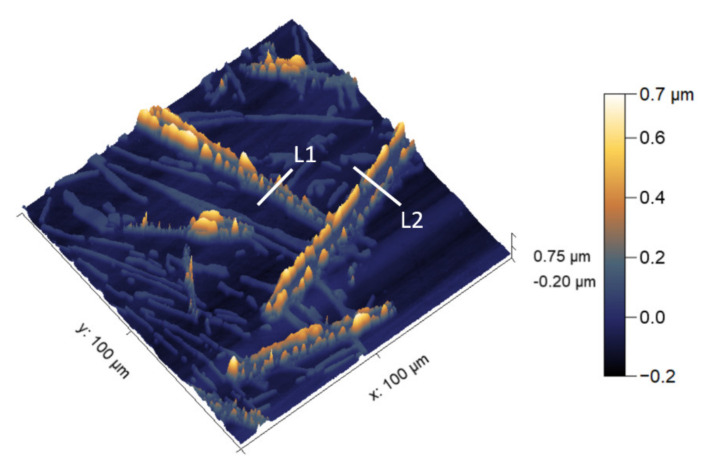
Atomic force microscope (AFM) map showing the Z-height of conversion-coated coarse microstructure substrate. L1 and L2 are line segments from which Z-height line profile was extracted for further analysis shown in Figure 8.

**Figure 8:**
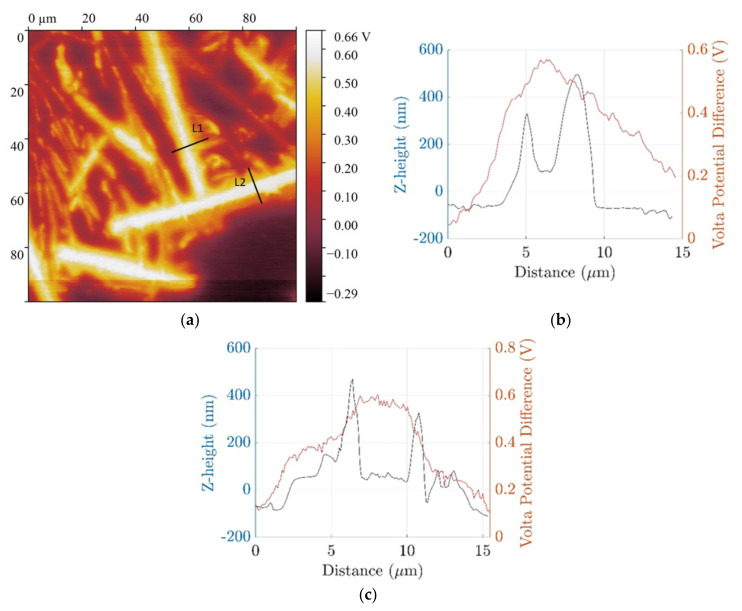
(**a**) SKPFM after 2 h deposition on coarse microstructure alloy; Z-height and volta potential profile of line segment (**b**) L1 (**c**) L2.

Another region of the microstructure where these localized deposits formed on the boundaries of the Fe-rich β IM was characterized with AFM-SKPFM and is shown in Figure 7 and Figure 8. The z-height of the localized deposit on the boundaries of the Fe-rich β IM was found to be in the range of 400–600 nm. Due to differential deposition along the width of IM, denoted with lines L1 and L2, different ΔV were measured. At the boundaries, the ΔV is less than in the central regions of the IM where heavy deposition was not observed. A reduced cathodic potential at the local regions of the IM where deposition occurred is due to physical barrier formation by conversion compounds. This correlates well with the general consensus that the mechanism of corrosion inhibition involves cathodic inhibition. The central regions of the IM showing higher ΔV make them active areas for further reduction of oxygen and consequently may result in subsequent local conversion compound precipitation.

In the case of small Fe-rich IM, localized depositions with a “cracked-mud” type morphology were not clearly observed under SEM, but Ce and signals from the locations of Fe-rich β IM were detected (Figure 7). The fine IM after etching are cathodically active and show the same ΔV as the coarse IM (refer Figure 3 and Figure 5 for ΔV_Fe_ of coarse and fine microstructures, respectively). Based on this argument that the fine IM are cathodically as active as coarse IM, they should theoretically support the reduction of oxygen and form localized deposits. Some unusually large IM in the fine microstructure (encircled in Figure 7) did, however, show the same type of localized deposit initiation on the boundaries of the IM. Si particles, as seen before with coarse microstructure, did not participate in the localized conversion coating deposition reaction.

### 3.3. Mechanism of Deposition on Fe-Rich Intermetallic Particles (IM)

**Figure 9:**
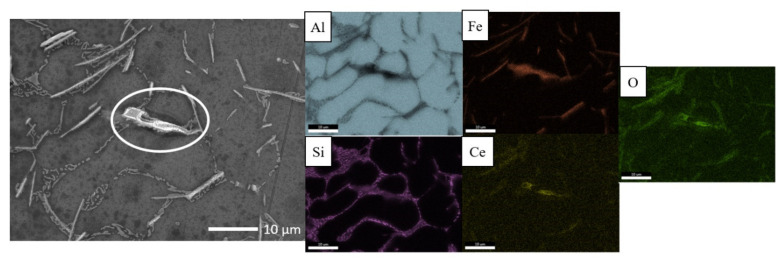
Two-hour 0.1 M CeCl_3_ immersion of fine microstructure alloy.

The conversion compound precipitate initiation sequence on Fe-rich β, hitherto, has not been reported. The Fe-rich intermetallics have lower reactivity and thus the localized CeCC deposition kinetics are slow. Through conversion coating treatment for a short time of 2 h, we were able to capture the initial deposition sequence on both coarse and fine microstructure Al–7Si–1Fe alloys (Figure 6 and Figure 9, respectively). Our observations of a border-type deposition on coarse Fe-rich IM and a faint deposition on fine Fe-rich IM allow the possibility to propose a deposition mechanism.

**Figure 10:**
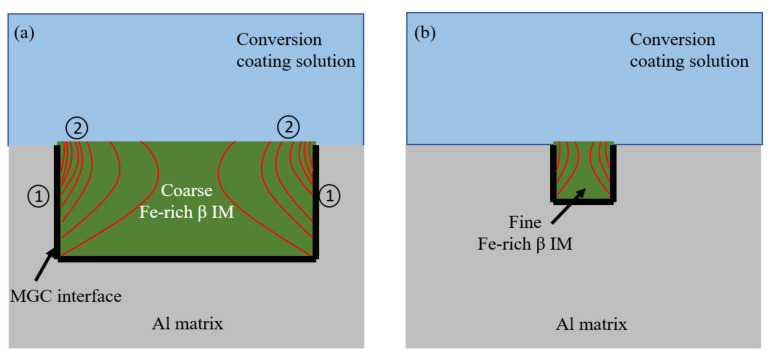
(**a**) Proposed explanation for differential deposition rates at different regions of a coarse Fe-rich β IM and (**b**) light deposition on a fine Fe-rich β IM. ① and ② denote interfacial reactions shown above this figure.

Observations show that a higher pH gradient exists over the border of the coarse intermetallic than on the central regions. It appears a greater number of electrons supplied from the Al-matrix (Reaction 1) during the active micro-galvanic coupling (MGC) reach the border of the Fe-rich β IM compared to that reaching the inner regions. Figure 10a visualizes the electron path through red lines in coarse intermetallics and shows a greater concentration of electrons over the intermetallic nearest to the micro-galvanic couple interface. Such a gradient in electron density at different regions of the intermetallics is hypothesized due to the presence of Si in the composition of β IM. The resistivity of Si is 6.4 × 10^2^ Ωm compared to that of Al and Fe which are 2.8 × 10^−8^ Ωm and 1.0 × 10^−8^ Ωm respectively. The presence of Si greatly increases the resistivity of the IM as compared to only pure Al or Fe components, leading to less electrons (generated at the micro-galvanic couple interface) reaching the central regions of the intermetallic (Figure 10a). The lower reactivity of Si-containing phases due to their lower conductivity has been demonstrated in a study by Zhu et al. [33]. Since electrons support the cathodic reduction of oxygen, which is the source for localized pH increase, in turn, needed for the precipitation of CeCC, this model proposes that differential deposition rates at the border of the coarse intermetallic compared to the central regions is due to the higher resistivity of the Fe-rich β IM.

Reaction ①: Al → Al^3+^ + 3e^−^

Reaction ②: O_2_ + 2H_2_O+ 2e^−^ → H_2_O_2_ + 2OH^−^ or O_2_ + 2H_2_O+ 4e^−^ → 4OH^−^

In the case of fine Fe-rich β IM, due to their very small size, fewer electrons are generated from the anodic reaction. Lower concentration of OH^-^ ions generated from oxygen reduction reaction may lead to faint precipitation of cerium compounds, which are not seen by SEM but are detected by EDS (Figure 9). Furthermore, due to their sub-micron width, the electron concentration gradient (red lines in Figure 10b) in such fine Fe-rich β IM is not as pronounced as in the case of coarse IM (whose width can be a few microns).

The deposition of conversion compound precipitates on Cu-rich IM, in contrast, appeared to cover the whole IM even after short immersion times [22]. Cu is a good conductor of electricity and thus provides an excellent path to the passage of electrons with a very low electrical resistivity of 1.7× 10^−8^ Ωm. This leads to a rapid and uniform transfer the electrons from the MGC interface to the IM–liquid interface allowing a sufficiently high pH gradient for spontaneous deposition of conversion compounds.

## 4. Conclusions

The paper focused on investigating Ce-based coatings deposition mechanism on heterogenous Al alloys microstructure not containing Cu. It was proved that Cu is not necessary to induce ceria deposition triggered by the microstructure electrochemical activity. Moreover, the following conclusions can be summarized from the study:

The coarse and fine IM in the polished condition showed different volta potential difference, but after etching with NaOH solution, became the same value.

Preferential heavy deposits initiated on the border of the coarse Fe-rich IM and light deposition on the fine Fe-rich IM was observed after subjecting the alloys to conversion coating treatment by immersion in a 0.1 M CeCl3 solution for a short period of 2 h.

No SEM-EDS visible deposition was observed on Si particles in both the microstructures.

A mechanistic explanation for the preferential heavy deposition along the border of the coarse Fe-rich IM is proposed based on its low conductivity due to the presence of Si in its composition.

## Figures and Tables

**Table 1:** Selected composition of the model alloy.

Element	Al	Si	Fe
Weight %	Balance	7	1

## Data Availability

The article contains the necessary data.
